# Application of PRECEDE-PROCEED Planning Model in Transforming the Clinical Decision Making Behavior of Physical Therapists in Myanmar

**DOI:** 10.3389/fpubh.2019.00114

**Published:** 2019-05-08

**Authors:** Phyu Hnin Hlaing, Patricia E. Sullivan, Pakaratee Chaiyawat

**Affiliations:** ^1^Faculty of Physical Therapy, Mahidol University, Bangkok, Thailand; ^2^Mahidol-Oxford Tropical Medicine Research Unit, Faculty of Tropical Medicine, Mahidol University, Bangkok, Thailand

**Keywords:** clinical decision making, Myanmar physical therapist, educational workshop, PRECEDE PROCEED model, curriculum development, Behavior Changed Intervention

## Abstract

**Introduction:** Physical therapists in Myanmar use a prescriptive model of Clinical Decision Making (CDM). Improving CDM effectiveness is one essential factor in professionalizing practice and enhancing patient outcomes. This study assesses the changes in CDM skills and behaviors using the PRECEDE-PROCEED planning Model (PPM).

**Methods:** In the PRECEDE planning phases, we investigated the current clinical decision making knowledge, and process, clinical practice culture, and contributing factors of CDM among Myanmar physical therapists. A qualitative approach consisted of 18 in-depth interviews and one focus group discussion was used. In the PROCEED evaluation and implementation phases, we developed and presented the CDM educational book at CDM workshop, which was a 4-day intensive program in Yangon, Myanmar with 34 participants. The participant's CDM knowledge and processes were assessed before and after the educational program to explore the potential impact on implementing CDM which can ultimately improve patient care in the health settings of Myanmar.

**Results:** In the PRECEDE phases, we explored the predisposing and reinforcing factors of Myanmar physical therapists' CDM. We found that CDM models and deliberative decision making process that is used internationally were not followed by Myanmar physical therapists who followed the physician's prescriptions. Teaching and learning emphasize a stimulus-response-repeat-outcome cycle without internal processing or application to clinical situations. Using the PROCEED model components, we developed a 14 chapters CDM workbook and a 4-day workshop as a behavioral change intervention. Participants' prior technical CDM behavior was transformed into professional CDM behavior that included an understanding of clinical practice models and improvement in the cognitive process of CDM processes. The workbook coupled with the intensive active-learning, hands-on workshop of examination and intervention procedures were effective in improving CDM.

**Discussion:** The application of PPM provided a through understandings of current CDM process of Myanmar therapists and aided in the development of the tailored CDM educational program to improve participants' CDM. Using the PPM model for developing a set of Physical Therapy educational content and curriculum was new. The application of PPM was beneficial to use accepted clinical practice models, standardized tests and measures, set goals and clinical outcomes, reassessed to determine change and implement evidence-based practice.

## Introduction

Myanmar is a developing country struggling for a brighter future currently at the stages of democratic transition Myanmar's healthcare system was ranked the second worst in the world by WHO (1). The quality of health care providers and the Quality Of Life (QOL) of Myanmar people have been affected from over fifty years of neglect (2) and isolation. Recently, Myanmar's economy is slowly improving, however, leading to the development of non-communicable diseases (NCDs) as the new epidemic amongst the affluent population. According to the WHO report, 68% of all deaths due to NCDs occurred in Myanmar in 2016 (3). Generally, for the lower-middle income countries, the treatment choice for NCDs depends on their economic status. Among the treatment choices for a wide spectrum of diseases, Physical Therapy has been increasingly demanded. For example, Physical therapists were in the frontline for the treatment of poliomyelitis which is an acute viral disease and results in a motor paralysis, followed by muscular atrophy and often permanent deformities (4). As the profession evolves and develops, physical therapists treat the patients to optimize function and ability for not only physical well-ness but also mental and social well-being to improve the quality of life (QOL).

In recent years, due to their involvement in widespread diseases, physical therapists have been increasingly demanded by the public. However, the role of Physical Therapy has been underestimated in the health care system and autonomy for Physical therapists have been minimal. Improving patient care is a priority goal for all healthcare professionals including physical therapists and this requires the achievement of effective and professional Clinical Decision Making (CDM) skills and behavior (5). The implementation of effective professionals CDM skills and behavior is a requirement of an autonomous practitioner (6, 7). The attainment of increased autonomy for physical therapists is a high priority to upgrade a profession to provide a quality service (8). Autonomous practice requires that the diagnosis of, interventions for, and prevention of impairments, activity limitations, and participation restriction. They must take into account the environment and personal barriers related to movement, function, and health (9). Attaining this level of decision making skill is one of the goals of this study.

In the physical therapy profession globally there is a growing body of literature that describes different approaches and strategies to improve CDM skills (5, 10–12) and the factors influencing decision making (13–15). Continuing professional education, one of the factors influencing CDM, is a critical component in the development of CDM skills which has been lacking in Myanmar. In Myanmar, physical therapists practice from a prescriptive model providing the treatment instructed by the referring clinician, rather than using analytical CDM skills based on accepted clinical practice models. Furthermore, therapists in Myanmar have had limited outside influences which narrows their knowledge of practice models such as the International Classification of Functioning, Disability, and Health (ICF) (16). There is a paucity of published literature on improving Myanmar physical therapists' CDM skills and behavior, or in other developing countries (17).

An additional effort is required to develop a theory-based intervention to upgrade professional physical therapists' skills and behaviors. In this study, we used the PRECEDE-PROCEED Model (PPM) as a framework to design educational interventions (18, 19). The PPM was originally developed by Green and Kreuter (20) and has been used extensively in the field of public/community health education. It guides the user through a series of diagnostic processes leading to the development of an evaluation and an intervention (21, 22). The application of the PPM in the physical therapy profession is new. In this study, we implemented the PPM model to design and implement the educational content to assess and subsequently improve the Clinical Decision Making (CDM) skills of therapists in Myanmar.

The main objective of this study was to explore the current level of CDM using PPM model among physical therapists in Myanmar so as to develop, implement, and test the educational program intended to improve the decision making behavior and their practice.

## Materials and Methods

**Figure 1:**
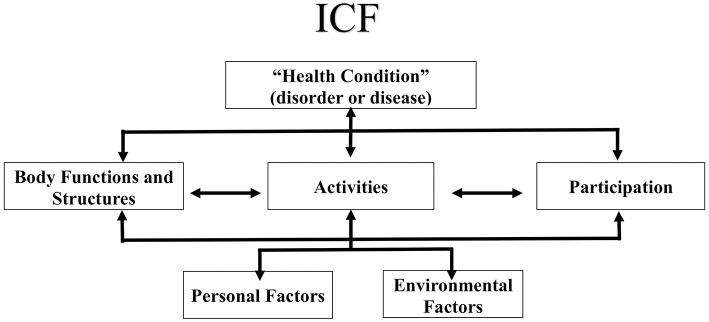
The Framework of International Classification of Functioning, Disabilities and Health (ICF). © World Health organization (WHO), 2019. All rights reserved (reprinted with permission from the World Health organization).

PPM was used as an overarching framework to determine the need of a CDM educational program (Figure 1). Two groups with the same characteristics participated in this study. With the first group, we assessed the CDM skills, identified the influencing factors and explored the routine clinical practice of Myanmar's Physical Therapists. The PROCEED phase of PPM was applied to develop the CDM educational program which was evaluated with the second group. We modified and adopted the phases of PPM. We have conducted this study according to the protocol approved by Mahidol University Central Institutional Review Board (MU-CIRB) with protocol number: MU-CIRB 2016/169.1810. We also had the ethical approval from the Department of Medical Research, Ministry of Health and Sports, The Government of the Republic of the Union of Myanmar with the approval number: Ethics/DMR/2017/035.

### Planning CDM Educational Program Based on the PRECEDE Planning Model

In the first three phases of modified and adopted PRECEDE phases (Table 1), we reviewed two sources of information: (1) the literature: how experienced therapists practice clinical decision making, what type of CDM models are used to improve decision making, the differences in its process (between novices and experienced physical therapists), and the factors influencing the CDM processes; and (2) the Myanmar physical therapist's clinical decision making process. We identified gaps between Myanmar physical therapist's current practice and the intended outcomes of the CDM process by investigating their CDM knowledge and process. To achieve the objective of the gap analysis, the qualitative approach, an in-depth interview was conducted with 18 Myanmar Physical Therapists. The inclusion criteria were (1) at least 2 years of clinical experience, and (2) practicing in Myanmar at the time of the study. The exclusion criteria were (1) participants who were not able to understand the instructions in English and (2) participants who were not graduated Physical Therapy program in Myanmar. More than 20 participants were available out of 30 participants who were invited and the interview was stopped at 18 because of data saturation (Table 2). All the invited participants were provided the consent form and the study information sheet where the detail study procedures are thoroughly documented. One CDM expert and one qualitative research expert provided guidance. An interview guideline (Table 3) was prepared in advance based on the literature. Participants were asked about their decisions to determine the underlying problems, choose the tests and measures to use, consider the environmental factors, and determine the treatment plan and discharge. The interview sessions were audio-recorded. A brief introduction included the general purpose of the assessment and planning, the role of the interview, the approximate time required for the interviews, and the issues around confidentiality and anonymity. After the initial interview, the interviewer followed up with some of the participants by e-mail or Facebook messenger to complete additional information based on the interview guide.

**Table 1:** Modified PRECEED phases of PPM.

**PPM Phases**	**Adopted PPM in developing CDM educational material**	**Participants**	**Methodology**
Phase 1 and 2- social and epidemiological assessment	Social assessment and literature review	18 Myanmar PTs with the inclusion criteria of: (1) at least 2 years of clinical experience and (2) participants practiced in Myanmar at the time of joining the study.	(1) The literature review (2) In-depth Interview and Focus Group Discussion (Qualitative approach)
Phase 3: Behavioral and environmental assessment	Assessment of myanmar PT's CDM skills		
Phase 4: Educational and ecological assessment	Investigation of physical therapy education in myanmar and identifying the influencing factors of myanmar PT's CDM		
Phase 5: Administrative and policy assessment	Exploring routine clinical practice of Myanmar's therapists		

**Table 2:** Participants characteristics for in-depth interviews.

		**Participants (*n* = 18) (%)**
Age	20–25 years	28
	26–32 years	50
	33–38 years	5
	Above 39 years	17
Practicing area	General	83
	Pediatrics	11
	Disability	5
Workplace	Private practice	12
	Yangon general hospital	55
	University of medical technology clinic	5
	Yangon orthopedic hospital	28
Years of experiences	2–4 years	39
	4–8 years	33
	>8 years	28
Government staff	Yes	39
	No	50
	Retired	11
Status	Clinician	83
	Demonstrator	11
	Community therapist	5

**Table 3:** Interview guideline.

“How and when do you make clinical decisions?” • When you first see a patient what do you do to determine what is wrong with them and how you will treat them? • If you use tests to measure range or strength or function how you choose which ones to use? • Do you ever consider any other factors such as medical, family or work that might be related with patient's health condition? • How do you decide how to treat all the problems the patient may have? • What options do you think about when deciding how to treat them? For example, does the patient need a brace, education, and home modifications? • How much confidence do you have that your intervention will improve the patient's problem? • How do you know the patient is improving? • How often do you assess whether the patient is responding as you would expect? • How do you decide when to discharge the patient? • How often do you think about the decisions you make?

Data analysis for In-depth Interview: The interviews were fully transcribed and analyzed using content analysis. All participants were assigned a code to ensure anonymity in the transcript (7). While doing content analysis, we had to ensure stability which refers to the tendency for the coder to code the same data in the same way over a period of time. The content analysis was conducted by 4 steps including identifying the categories, comparing and contrasting the various major and minor categories, reviewing the categories and finally returning to the original transcripts to ensure that all the information that needed to be categorized had been so (23). The triangulation method established with the supervisors of the project checked the integrity of the inferences drawn (24).

In phase 4, we identified the predisposing, reinforcing and enabling factors of Myanmar physical therapists' behavior, knowledge, and attitudes on clinical decision making based on the data from a content analysis of 18 in-depth interviews. We addressed the following questions “What are the predisposing factors for Myanmar PTs that provide motivation for the current behavior, knowledge and attitudes on CDM?” “What are the reinforcing factors to continue the routines of current CDM process?” and “What could be the enabling factors for Myanmar PTs to realize to make a change in their CDM skills or what professional activities encourage them to motivate to make a professional improvement?” Researcher categorized the interview responses into three factors: predisposing factors, reinforcing factors, enabling factors using an iterative process of content analysis. In phase 5 of planning CDM educational program, we carefully examined the information from previous phases and identified the background CDM knowledge of Myanmar therapists and analyzed the CDM content and process knowledge gaps. As an outcome, we designed the 4-day intense workshop and the 14 chapters of Clinical Decision Making workbook.

### Conducting CDM Workshop and Evaluating the Program Based on PROCEED Planning Model

**Figure 2:**
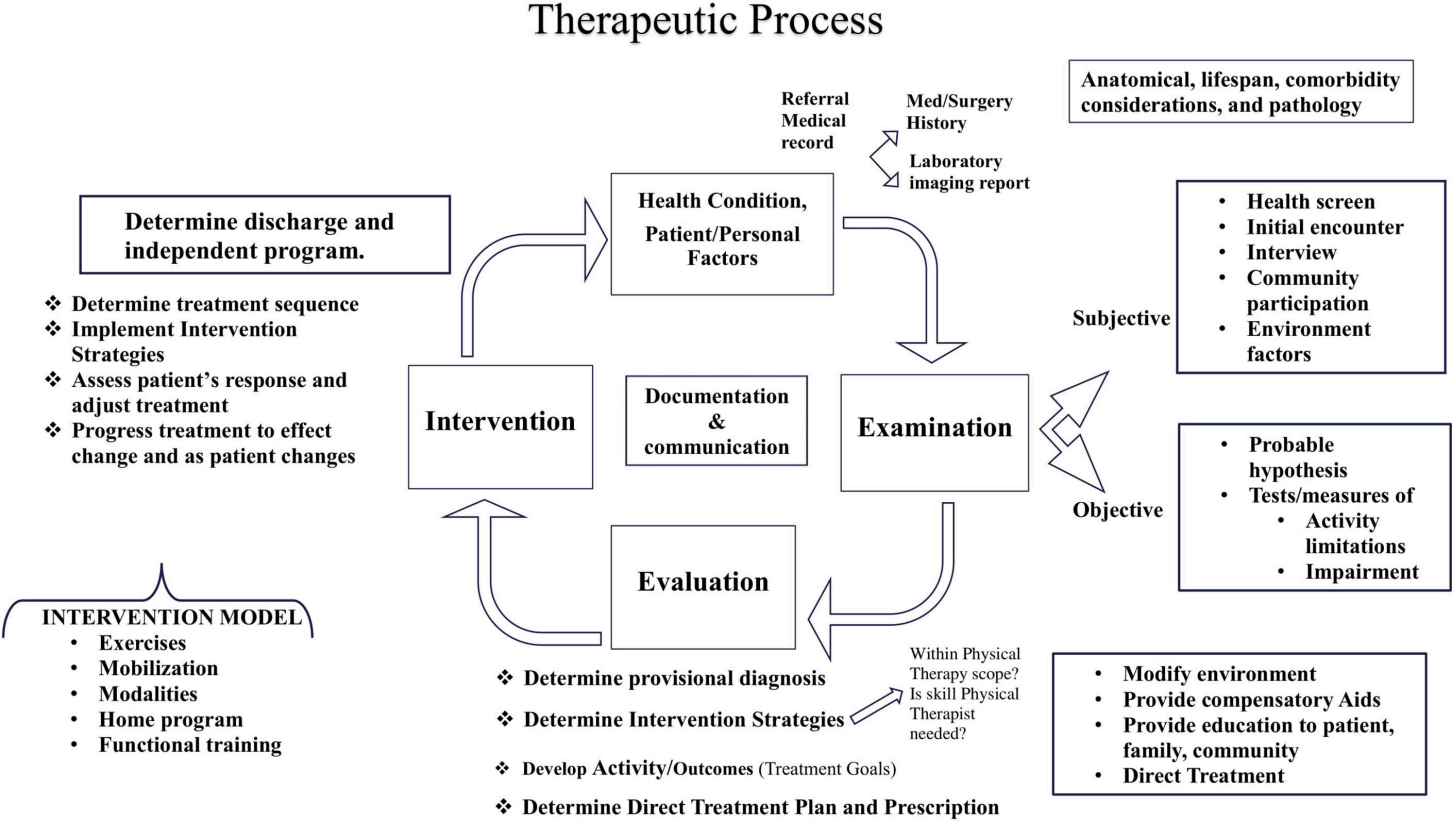
Therapeutic process.

**Figure 3:**
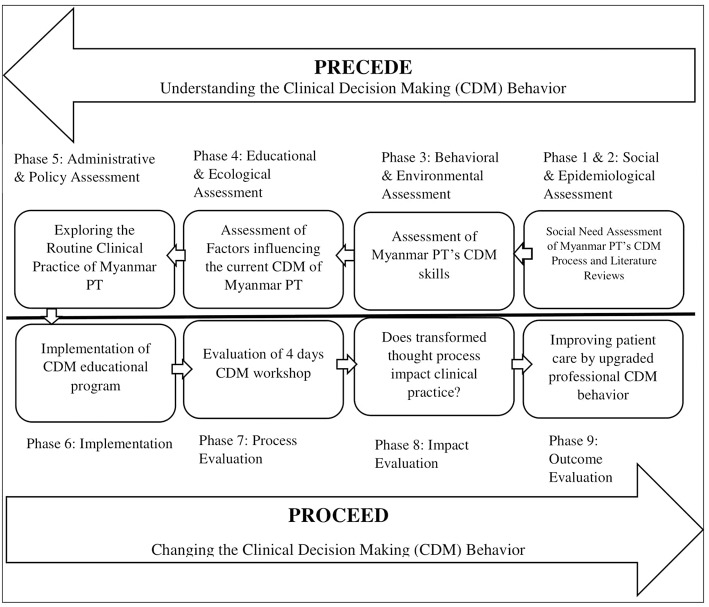
Applied PPM model for CDM behavior transformation.

Using the modified and adopted PROCEED planning model (Table 4), we developed the workshop and workbook as the educational intervention to improve CDM skills. The CDM educational content is based on two clinical practice models: The International Classification of Functioning, Disability and Health (ICF) (Figure 2) and Therapeutic Process (TP) (Figure 3) (25), a clinical model which sequences the patient through admission to discharge and is the expansion of Patient Client Management Model of American Physical Therapy Association (26).

**Table 4:** Modified PROCEED phases of PPM.

**PPM Phases**	**Adopted PPM in developing CDM educational material**	**Study participants**	**Methodology**
Phase 6: Implementation	Development of the CDM educational content and 4 days CDM worksh	34 Myanmar PT with the similar inclusion criteria from the PRECEDE phases of planning model	Evaluated 4 days intensive CDM workshop by applying CDM assessment worksheet before and after the workshop. (Mixed method approach).
Phase 7: Process Evaluation	Evaluation of 4 days CDM workshop		
Phase 8: Impact Evaluation	Does transformed thought process impact clinical practice?		
Phase 9: Outcome Evaluation	Improving patient care by upgraded professional CDM behavior		

The ICF, developed by the World Health Organization (WHO) is a framework that organizes and can be used to document information about a person's health condition, their functional activity, ability and the contextual factors influencing health. The ICF conceptualizes functioning as a dynamic interaction between a person's health condition, and their environment and personal factors as well as the physical factors that can contribute to the patient's activity limitation and community participation restriction (16). The physical therapy profession has adopted the ICF as a framework to conceptualize functioning from a holistic approach and has suggested that the ICF be used to plan examinations and interventions targeting the whole individual and their environment (27). The TP describes the phases of clinical decision making as patient-health condition-information, examination, evaluation, intervention, and discharge. Combining the TP with the ICF, the clinician describes the health condition as the primary and secondary diagnosis, examines the patient's movement characteristics (ROM, strength, balance, etc.) and their level including ambulation and activity of daily living. Then evaluate the findings, develop the goals to improve the impairments and implement the treatment plan (28) (Figure 2).

The CDM interventions represent the knowledge within the four main elements of Clinical Decision Making process: (1) knowledge of health conditions and comorbidities, (2) examination of physical systems and contextual factors, (3) evaluations of the examination findings, setting goals and outcomes, and determining a plan of care, and (4) intervention to achieve treatment goals and functional outcomes. These four elements represent the sequence of the educational content of the workshop and workbook (Table 5). The content was reviewed and the final draft formatted with the help of a CDM expert.

**Table 5:** CDM workbook chapters and objectives.

**Day**	**Chapters**	**Objectives**
Day 1	Chapter 1–4	**Chapter 1: Health condition** • Identify the ICF and Therapeutic Process (TP) • Within the ICF identify IMP and AL associated with a person having fallen with a possible sprained ankle • Consider PF and EF • Diagram different categories of the ICF **Chapter 2: Progress from health condition to Examination** • Progress from the patient condition to examination in the Therapeutic Process • Identify IMP and AL commonly associated with a sprained ankle using the ICF model • Identify specific measures for each anticipated IMP and AL • Describe the influence of pain on the reliability and validity of other IMP and AL tests. • Project anticipated changes in IMP and AL over time **Chapter 3: Impairment and activity limitation** • Identify IMP and AL expected with a patient that has the HC of their patient • Identify, Reflect For, ICF categories they have observed with the patient they have chosen • What is different between expected vs. observed findings? • What examination measures could have been used to objectify IMP and AL, How much specificity is needed? **Chapter 4: Examination** • Determine standardized tests and measures • Practice standardized tests and measures
Day 2	Chapter 5–8	**Chapter 5: Evaluation** • Analyze the balance impairment • Practice standardized tests (balance tests) • Determine provisional diagnosis **Chapter 6: Contribution factors and impairment change over time** • Determine contribution of PF and EF • Determine anticipated changes of impairment overtime **Chapter 7: Activity outcomes and treatment goals** • Determine activity outcomes and treatment goals **Chapter 8: Intervention strategies** • Determine the intervention strategies for your patient • Develop the direct treatment plan • Discuss intervention model
Day 3	Chapter 9- 11	**Chapter 9: Review guidelineChapter 10: Intervention** • Determine the initial priorities of treatment • Develop a plan of how to achieve the treatment goals and activity outcomes • Practice therapeutic procedures to improve mobility **Chapter 11: Intervention Model**
Day 4	Chapter 12- 14	**Chapter 12: Therapeutic Procedures** (Intervention procedures according to the Intervention Model) **Chapter 13: Home exercise and community care** **Chapter 14: Your whole patient**

We conducted 4 days CDM workshop in March 2017 at University of Medical Technology, Yangon. A total of 34 participants with similar characteristics of PTs from the in-depth interview attended the CDM workshop, the average age of the participants was 29 and all participants (100%) were female. All participants signed an informed consent form describing the benefits and risks of the study. The workshop began with the first assessment for ~30 min. Participants were assigned a number. They put the number on the top of the assessment worksheet to protect their identity. A demographic data sheet was given during the workshop registration. Participants came from 17 different hospitals and clinics across Yangon, Mandalay and Shan state of Myanmar and their duration of clinical practice ranged as follows: 2–4 years (52.5%), 4–8 years (15.3%) and more than 8 years (32.2%). Their area of practice was categorized in four areas; Musculoskeletal (30.5%), Neurology (6.8%), Pediatrics (18.6%), and General (44.1%). The sample size was calculated according to the study design of pretest and posttest for the same subject. We would need 27 samples to see the average change in knowledge from pretest and posttest with the alpha 0.05, power 0.08 and medium effect size of 0.5. However, we recruited 34 participants to cover 20% anticipated dropout. The participant recruitment was done by the involvement of authorities from the University of Medical Technology (UMT) (both Yangon and Mandalay) and Physiotherapy Department from Government hospitals in Myanmar. The CDM workshop proposal was sent to the Ministry of Health and Sports to obtain approval to conduct the CDM workshop at the UMT, Yangon.

During the workshop, participants were encouraged to actively participate by sharing and discussing the description of a patient they were currently treating in pairs then in a small group and then a big group. The workshop guided the participants through the CDM processes inherent in the two clinical practice models; (1) Therapeutic Process, a clinical model which sequencing the patient through admission to discharge and (2) the ICF, the WHO model describing the components of health and function and Also participants were encouraged to reflect on the choices they made or could make with the patient. The workshop followed the chapters in the workbook and was designed to complement and expand the workbook information.

We evaluated the CDM intervention by ensuring all participants completed the CDM assessment worksheet which was developed by an intense process of assessing each question for relevance and importance and determining the worksheets' psychometric properties. The CDM assessment worksheet has 3 types of questions: (1) nominal Yes/No, (2) ordinal Likert scale, and (3) qualitative data open-ended. In section-1, the participants were asked whether they asked the patient's past medical history, medication use, co-morbidities, contextual personal, and environmental factors and if they have used the standardized assessment tests. In the second part of the worksheet they responded to how often they asked the patient about personal factors (patient's literacy, patient's expectation in therapy), environmental factors (home and work situation, patient's support at home, patient's access to the treatment), co-morbidities or serious medical condition and, their reflection on the examination findings and the outcomes of the treatment. In section three, they were asked about their decision making thought process, the sequential thinking process of the therapeutic process from examination to discharge the patient. The CDM assessment worksheet was administered three times, once before CDM workshop, once immediately after the workshop and again 1 month after the CDM workshop.

Data analysis for CDM workshop: Non-parametric statistical analyses (Cochran Q test and Friedman's two-way analysis of variance) were applied to analyze the data from the CDM assessment worksheet to determine if the CDM workshop and the workbook changed the participant's decision making performance. The responses from open-ended questions were analyzed by content analysis of identifying categories, comparing and contrasting the various major and minor categories, and ensuring all the categories were correctly categorized by returning to the original answers. The eight open-ended interview questions were: (1) If the patient is complaining of pain how do you figure out what is causing the pain?; (2) What are the components of the ICF?; (3) How do you modify or progress the treatment to help the patient improve?; (4) How do you determine what combination of treatments you will use with the patient?; (5) As shown in the ICF what could be the factors influencing the possible outcomes?; (6) How do you know the patient is improving?; (7) What is the difference between examination and evaluation?; and (8) If the patient does not improve as you expect what do you do? In this phase, we also explored the gaps between what was planned and what spontaneously emerged in the CDM workshop and including the relations between the components of the whole CDM program.

From the one-month post-workshop data, we identified the retention effect of the transformed knowledge and the CDM processes thought processes and the possible impact on routine clinical practice. The original process in which decision making was dependent on the doctor's prescription with no additional examination or analysis was transformed to professional decision making behavior based on the examination of the patient's impairments and activity limitations and implementing a treatment plan based on that knowledge. Adhering with the PRECEDE-PROCEED Model we also explored the potential factors that could influence the long-term behavior change in the Myanmar physical therapy practice and its impact on improving patient care which is the ultimate goal of developing CDM educational content.

## Results

The first step of the PRECEDE process is a Social & Epidemiological Assessment in which we conducted a literature review and questioned therapists. The literature is clear that providing patient-centered treatment based on a thorough assessment of the patient's condition, their impairments and activity limitations with an understanding of their personal and environmental factors in the decision making process followed by experienced therapists (29, 30). Educational literature emphasized a student-centered, active learning approach in order for the student to internalize knowledge and successfully apply that knowledge (31). From the responses of therapists to the questionnaire, we found that most therapists do not perform a thorough examination but rather a cursory assessment based on the doctor's prescriptions.

In the Behavioral & Environmental Assessment phase we interviewed eighteen participants with at least 2 years of clinical experience to assess the clinical decision making processes of therapists, Following a thorough study of the transcriptions, the thematic content analyses indicated that the context units converged into ten categories: (1) Patient's first visit, (2) Assessment/examination, (3) Asking contextual factors, (4) Intervention, (5) Options in intervention decisions, (6) Confidence of intervention, (7) Knowing the patient improvement, (8) Reevaluation, (9) Discharge, and (10) Reflection for, during, and on patient care.

Based on the emerged themes, the researchers defined the current process used by the Myanmar physical therapists. These Myanmar therapists did not commonly follow a deliberative type of decision making process rather followed the physician prescribed treatment with minimal additional examination or evaluation. Standardized physical therapy tests and measurements were infrequently performed and retesting to assess change was uncommon.

“*I follow the prescription and what the senior decides for the treatment*” one participant reported (P-3).

Beyond direct treatment, therapists did not determine the need for or implement other intervention strategies including education, providing compensatory aids, or modifying the environment.

“*Advice on posture awareness should be one of the options in treatment but we don't usually encourage the patient to modify their home or workplace as we think it's not affordable by them*” (P-12).

The less experienced therapists more commonly followed the prescribed treatment with no modification.

In Phase 4 the Educational & Ecological Assessment, we found that the physical therapy education process is a major predisposing factor for current knowledge and attitudes on CDM. Participants indicated that they received lectures as a major portion of their education. There could be many factors influencing the teacher's teaching styles and the student's learning styles. In Myanmar, the student-centered classroom is not implemented. Students acquired new knowledge by memorizing the presented information. The students fail to link this content information with real-life situations and do not have the opportunity to mentally process this new knowledge. There are a few collaborative student's activities in the classroom. Students do not control their own learning. They respond with the predetermined right answers rather than discussing the advantages and disadvantages of a range of options.

During Phase 5 the Administrative and Policy Assessment we found that in the clinical practice setting, critical thinking based on evidence and on reliable information is not encouraged. The deep-rooted culture of strictly following the physician's intervention prescription as well as other challenges, such as limited time for examination was found as the reinforcing factors for Myanmar PT to continue the routines of current lack of CDM process.

Combining the evidence from the literature, from the PRECEDE phases of participant interviews, and from the assessment of the educational and social milieu we determined the factors that needed to be emphasized in the development of CDM educational content. We then designed the implementation: the 4 day CDM workshop and was evaluated through PROCEED phases. The CDM educational content provided the therapists with the knowledge, and enabling skill for Myanmar PT to make a change in their CDM and clinical practice.

We found that participants adherence to internationally accepted norms of CDM processes improved on the post-workshop CDM including the retention of the concepts at 1 month. Participants after the intervention more routinely asked the patient's past medical history, medication, and comorbidities. They took patient's information using standardized tests and asked about the contextual personal and environmental factors as assessed in section 1 of the assessment worksheet. The Cochran's Q test demonstrated statistical differences in the proportion of participants whose routine patient examinations changed over time, χ^2^(2) = 62.312, ρ < 0.0005. Participants were asked how often they asked personal, environmental factors, consider co-morbidities as described in the ICF and including the reflections on their own practice. A significant difference was found between the baseline pretest and immediate and one-month posttest, Chi-square = 251.9, *p* < 0.001.

The qualitative analysis of the eight open-ended questions was analyzed by content analysis. Before the CDM intervention, participants reported that in the examination phase, the pain was the impairment that Myanmar therapists addressed first when treating the patients. After the workshop, almost all of the participants mentioned that they include subjective examination about the health condition, community participation and personal and environmental factors.

“*If the patient is complaining of pain, I would figure out what is causing the pain by patient's gesture, posture, expression and initial encounter, and examination which is a subjective and objective examination.”* (P-02)

In the pre-test, 64% of participants indicated knowledge of the ICF practice model in comparison to 100% after the workshop. After the workshop, therapists could group the examination findings into ICF categories and implement the intervention with greater confidence in achieving desired outcomes.

“*I would determine the combination of treatment according to impairment resulting from activity limitation”* (P-24)

Regarding the Intervention phase of clinical decision making process, after the workshop, participants recalled intervention strategies of modifying the environment, providing compensatory aids, providing education to patient, family, community, and directing treatment. These strategies of intervention were not included in the pretest.

“*I would check the co-morbidities, other health condition, find problems in home conditions and environment situation etc.”* (P-12) “*I would also recheck the co-morbidities, and if this is beyond the scope of physiotherapy, I'd rather refer to physicians”* (P-05)

## Discussion

This study demonstrated that using the PPM model to develop and implement an educational intervention for physical therapists was successful. Following this model, we identified the predisposing factors of traditional lecture-based PT education, current Myanmar PT's practice culture including the policy and time and the emotional capabilities of physical therapists, for instance; confidence and self-efficacy. The implementation phase of PPM helped in designing the CDM educational intervention which can contribute to PT formal education transformation in Myanmar. Following the PPM based CDM intervention which was tested as one of the enabling factors helped participants understand the need of practicing guided CDM process efficiently in the clinical practice is crucial in improved patient care and also motivated them to make a change in their practice routines. Moreover, PPM based intervention equipped them with confidence and self-awareness as they were provided with a step-by-step CDM process guideline. Confidence and self-awareness as predisposing factors are a facilitator for improving emotional capabilities of decision makers. Therefore, those factors are important factors in establishing and maintaining effective relationships in the workplace with the patients. PPM model also identified the reinforcing factor of following the prescriptive medical model. The study also identified the CDM knowledge gap regarding the ICF and Therapeutic Process (TP) practice models which are central in improving CDM. We then designed and implemented a CDM intervention. By using the PRECEDE-PROCEED model processes we showed improvement transforming the thought processes and reported clinical practice of Myanmar therapists. The thought process of sequential thinking of CDM from the examination phase to discharge phase of the patient is acquired. The new way of reasoning or decision making guided the participants with a holistic approach to the patient in every phase of CDM. In the examination phase, they obtained a history, performed relevant systems review, and selected and administered the specific tests and measures. In the evaluation phase, they made clinical judgments based on data gathered during the examination then they labeled a disorder, syndrome, category of impairments in body structures and function, activity limitations, or participation restrictions. In the intervention phase, they provided purposeful and skilled interaction with the patient, including coordination, communication, documentation, patient-related instruction, and procedural intervention. The findings of transformed thought processes after CDM workshop suggested the effectiveness of the 4 days PPM based CDM workshop. The findings of this study demonstrated that assessing the current social and clinical context in which therapists provided care and subsequently designing and providing a step-by-step, sequenced guideline and active learning workshop changed their reported patient interventions.

An understanding of the participant's current CDM process, their culture, their educational background, and the influencing factors enhanced the overall participant improvement. Myanmar therapists do not commonly follow the deliberative type of hypothetico-deductive clinical decision making approach. Hypothetico-deductive reasoning process involves cue recognition, hypothesis generation, cue interpretation and hypothesis evaluation (32). Prior to the intervention they only followed the prescription without any additional examination or evaluation. Performing standardized tests and measurements and developing a treatment based on this information was uncommon.

Interestingly the result of this study indicated that equipping the participants with the resources, guidance and evidence of CDM process improved their knowledge of CDM process and transformed their thoughts. Studies suggest that equipping with educational methodologies tools is important to acquire new predisposing, reinforcing, and enabling factors (33, 34). Teaching CDM as provided by the workshop bridged the gap between Myanmar physical therapist's CDM knowledge and international standard as described in the literature by transforming their thought process to make more effective clinical decisions and improve patient care. In addition, practicing different ways of thinking about patient care changed their beliefs, perceptions and importantly boosted their confidence in their choices in order to enhance patient care.

This is the first study reporting improving clinical decision making specific to the profession of physical therapy in Myanmar. The findings of the study suggested that knowing the knowledge about CDM process, the predisposing factors of Myanmar PT's behavior, knowledge and attitude on CDM is important. Similarly knowing the common routine and culture of clinical practice and the reinforcing factors is crucial to implementing a successful intervention.

The successful intervention could serve as a model to improve PT education in Myanmar and in other developing countries. The workbook and workshop encouraged active participation and reflection for, in and on practice. This student-centered educational approach is greatly different from the typical process in Myanmar.

What we do not know is how the participants shared their new knowledge in their practice settings. One study showed that once having been exposed to the concept, individuals continued to gain knowledge and understanding (33). Studies showed that a reason for changing behavior in continued learning could be the specific behavioral change teachings carried out in the training (33, 35).

This study suggested that our PPM based CDM educational content significantly enhanced participant's clinical decision making process that could ultimately improve patient care. This is the first study that identified the PPM's variables, predisposing, reinforcing and enabling factors of CDM from in-depth interview to develop the educational intervention and to be tested with physical therapists.

The application of the PRECEDE assessment phase and PROCEED planning and implementation phase was beneficial in the improvement of CDM skills and behaviors by gaining and understanding of the clinical decision making process as a gap assessment and development of CDM educational program. Although using the PPM model for developing Physical Therapy educational content (curriculum) is new, it has been widely used in public health education to design, implement and evaluate the health behavior change program (33–36). Applying a PPM model in developing a clinical decision making skills was appropriate to develop improved CDM skills needed for a behavioral transformation.

## Conclusion

Physical Therapist's technical decision making behavior can be transformed to professional clinical decision making behavior by applying PPM. First by knowing what their CDM process is and second, filling the knowledge and process gap by providing intense step-by-step structured PPM based CDM education as a behavioral change intervention. Although many changes are needed to upgrade the quality of patient care by physical therapists in Myanmar, one successful change was to achieve professional clinical decision making behavior. Participants were motivated to implement a more effective clinical decision making practice because of their success in the workshop. Participants benefited from being provided CDM process instructions which encouraged them to think autonomously to transform their thoughts process regardless of the common practice and routine of their clinical decision making practice.

### Implications for PT in Myanmar

The effective CDM educational intervention can be implemented in Myanmar as a Clinical Decision Making Curriculum at the Department of Physiotherapy, University of Medical Technology. The educational implementation process in Myanmar could demand tremendous commitments and works especially when it comes to working with the government bodies and policymakers. Although we recognized that bridging the gap between research and practice takes time and effort, the resources such as CDM interventions designed by this study are highly effective. In addition, therapists from other countries who are taught and practice in the manner similar to that of the therapists in Myanmar may also benefit from this type of assessment and intervention.

### Recommendations for Future Research

A further study could compare between individuals with different clinical experiences throughout the process of CDM thoughts transformation using the PROCEED phase. This study has highlighted the need for further investigation such as how learned CDM knowledge could transform their CDM behavior in a different clinical practice area. Similarly, future study to compare the behavior change by health education and by clinical practice can be helpful. Future studies can test the current intervention in different context and settings. Challenges of implementation can be furthered up by using models in implementation research. An investigation of the more in-depth relationship between CDM process and patient's treatment outcome is crucial for future research for more refined understandings.

## Ethics Statement

This study was carried out in accordance with the recommendations of the Mahidol University Central Institutional Review Board's Guideline with written informed consent from all subjects. All subjects gave written informed consent in accordance with the Declaration of Helsinki. The protocol was approved by Mahidol University Central Institutional Review Board (MU-CIRB), Protocol Number: MU-CIRB 2016/169.1810 and also approved by the Department of Medical Research, Ministry of Health and Sports, The Government of the Republic of the Union of Myanmar, Protocol Number: Ethics/DMR/2017/035.

## Author Contributions

PH mainly analyzed and interpreted all data and PC helped in analysis. PH was a major contributor in writing a manuscript and PS revised it. All authors read and approved the final manuscript.

### Conflict of Interest Statement

The authors declare that the research was conducted in the absence of any commercial or financial relationships that could be construed as a potential conflict of interest.
